# Gut microbiota: A novel and potential target for radioimmunotherapy in colorectal cancer

**DOI:** 10.3389/fimmu.2023.1128774

**Published:** 2023-01-31

**Authors:** Hanghang Yuan, Ruirui Gui, Zhicheng Wang, Fang Fang, Hongguang Zhao

**Affiliations:** ^1^ Department of Nuclear Medicine, The First Hospital of Jilin University, Changchun, China; ^2^ National Health Commission (NHC) Key laboratory of Radiobiology, School of Public Health, Jilin University, Changchun, China

**Keywords:** colorectal cancer, radioimmunotherapy, gut microbiota, fecal microbiota transplantation, probiotics

## Abstract

Colorectal cancer (CRC) is one of the most common cancers, with a high mortality rate, and is a major burden on human health worldwide. Gut microbiota regulate human immunity and metabolism through producing numerous metabolites, which act as signaling molecules and substrates for metabolic reactions in various biological processes. The importance of host-gut microbiota interactions in immunometabolic mechanisms in CRC is increasingly recognized, and interest in modulating the microbiota to improve patient’s response to therapy has been raising. However, the specific mechanisms by which gut microbiota interact with immunotherapy and radiotherapy remain incongruent. Here we review recent advances and discuss the feasibility of gut microbiota as a regulatory target to enhance the immunogenicity of CRC, improve the radiosensitivity of colorectal tumor cells and ameliorate complications such as radiotoxicity. Currently, great breakthroughs in the treatment of non-small cell lung cancer and others have been achieved by radioimmunotherapy, but radioimmunotherapy alone has not been effective in CRC patients. By summarizing the recent preclinical and clinical evidence and considering regulatory roles played by microflora in the gut, such as anti-tumor immunity, we discuss the potential of targeting gut microbiota to enhance the efficacy of radioimmunotherapy in CRC and expect this review can provide references and fresh ideas for the clinical application of this novel strategy.

## 1 Introduction

Colorectal cancer (CRC) is the third most common cancer in the world (1). Comprehensive data show that, globally, the incidence rate of CRC is 10%, and the mortality rate is 9.4%. The latest data shows that the average risk incidence rate and mortality of CRC among the middle-aged and elderly (50 years old and above) are declining, but it is worth noting that the incidence rate of CRC among young patients is increasing, which is the second common cause of cancer related death in the world (2). With the development of medical science and technology, the traditional treatment methods of CRC, such as surgery, radiotherapy, chemotherapy and other technologies, are constantly improving. Their combinations, such as surgery combined with postoperative radiotherapy, surgery combined with chemotherapy, are also gradually applied to clinical practice. However, not all CRC patients respond positively to treatment. Some patients may even have treatment related adverse reactions, local recurrence and distant metastasis. Therefore, it is very important to study new treatment methods or improve existing combined treatment methods for prolonging the survival period and improving the quality of life of CRC patients.

In the past few decades, immunotherapy has become one of the new options for cancer treatment. This new therapy mainly kills tumor cells by regulating or directly manipulating the patient’s own immune system. Compared with the traditional treatment of tumor, it has higher specificity, higher long-term survival rate and fewer side effects (3). For CRC, immunocheckpoint block (ICB) is the most common immunotherapy. However, “cold” CRC, which accounts for a large number of CRC patients, is not sensitive to ICB response. Studies have shown that the dMMR/MSI-H phenotype shows higher levels of ORR compared to the pMMR/MSS phenotype, which accounts for the majority. In addition, ICB increases the function of the immune system so that induce inflammatory side effects and induce immune-related adverse events (rash, colitis, diarrhea, hepatotoxicity, pneumonia, etc.) Therefore, how to improve the immunogenicity of “cold” CRC to improve the ability of the immune system to eradicate tumor cells is a recent research hotspot. What is exciting is that there is evidence that the organic combination of radiotherapy and immunotherapy can improve the sensitivity of CRC to immunotherapy, so as to enhance the ability of the immune system of CRC patients to kill tumor cells, resulting in an “1 + 1 > 2” effect.

It is worth mentioning that nowadays people generally believe that gut microbiota can support the overall health of human body by maintaining the integrity of intestinal structure and protecting the intestinal tract from pathogens. More and more studies have found that there is an unexpected association between gut microbiota and CRC. For CRC patients, it plays a role in the development, treatment and prognosis of cancer. More significantly, a growing number of studies have found a correlation between the gut microbiota and the treatment of CRC. In general, the gut microbiota tend to correlate with treatment efficacy and prognosis. For example, gut microbiota can influence the responsiveness to immunotherapy and the incidence of immune adverse events associated with immunotherapy in CRC. In the case of CRC radiotherapy, manipulation of the gut microbiota can also be used to promote sensitivity to radiotherapy and reduce radiation toxicity associated with radiotherapy.

In this review, we summarized several classical mechanisms of immunotherapy and radiotherapy in CRC, supplemented the latest research in the past few years, and discussed the feasibility and potential best combination strategy of radiotherapy and immunotherapy for CRC. More importantly, based on the important role of the gut microbiota in CRC immunotherapy and radiotherapy, we explored and reasonably speculated on the feasibility of the gut microbiota as a potential target and its roles in the combined radiotherapy, immunotherapy and radioimmunotherapy of CRC.

## 2 Gut microbiota—a potential therapeutic target for CRC

### 2.1 Microbiota landscope in CRC

Bacteria are an important component of the neoplastic microenvironment The bacterial-rich gut microbiota is known as the “forgotten organ” that affect many essential physiological processes in human body. The close relationship between alterations in the gut microbiota and CRC is widely recognized. Currently, the dominant flora in CRC progression remains undefined, but benefited from the development of microbiome profiling, including 16s rRNA and shotgun metagenomics, we have a more in-depth and comprehensive understanding of the taxonomic and functional characteristics of gut microbes and metabolites. A growing number of metagenomic and metataxonomic studies have revealed some potential anti-tumor probiotics and pro-carcinogenic microbiota.

Compared with healthy individuals, CRC patients have a lower abundance of protective probiotics and higher pro-cancer microbiota. A comprehensive analysis of the multinational microbiome using eight different cohorts of the CRC macrogenomic dataset found that: although the microbial species differed considerably in different groups, a subset of species with consistent alterations were identified, such as Alistipes onderdonkii, Parvimonas micra and Gemella morbillorum. A total of 48 bacterial species with elevated abundance were identified in CRC patients, including F. nucleatum, P. micra, Porphyromonas asaccharolytica, Desulfovibrio desulfuricans and Akkermansia muciniphila. Besides, protective species of butyrate-producing bacteria, such as Clostridium butyricum, Roseburia intestinalis and Butyrivibrio fibrisolvens, were reduced in patients with CRC compared to controls (4). Recent meta-analyses have ascertained cross-cohort microbial signatures associated with CRC, including enrichment for Clostridiaceae, Daniostoma, and Mycobacterium morganum (5, 6). Meta-analysis of 526 faecal shotgun metagenome datasets identified a microbial core of seven enriched bacteria in CRC, B. fragilis, a bacterium with enterotoxigenic capabilities associated with CRC, F. nucleatum, Parvimonas micra, Porphyromonas asaccharolytica, Prevotella intermedia, Alistipes finegoldii and Thermanaerovibrio acidaminovorans (7). **Table 1** summarizes the microflora that are increased and decreased in CRC patients compared to healthy individuals.

**Table 1:** Taxonomy summary of the above mentioned intestinal flora changes in the development and progression of CRC.

Increased intestinal microbiota	Reduced intestinal microbiota
Prevotella intermedia (8)	Bacteroidetes (9)
Gemella morbillorum (10)	Coprobacter fastidosus (11)
Desulfovibrio (12)	Bifidobacterium (4)
Enterococcus faecalis (12)	Butyrivibrio fibrisolvens (4)
Dialister pneumosintes (10)	Clostridium butyricum (4)
Alistipes finegoldii (7)	Roseburia (13)
Fusobacterium nucleatum (13)	Eubacterium (13)
Parvimonas spp (13)	Dorea (13)
Porphyromonas asaccharolytica (13)	Coprococcus (13)
Ruminococcus torques (10)	Faecalibacterium (13)
Akkermansia muciniphila (14)	Talaromyces islandicus (4)
Veillonella parvula (15)	Sistotremastrum niveocremeum (4)
Peptostreptococcus (13)	Macrophomina phaseolina (4)
Streptococcus gallolyticus (16)	Aspergillus niger (4)
Thermanaerovibrio acidaminovorans (7)	
Filifactor alocis (10)	
Escherichia coli (8)	
Campylobacter (15)	
Enterotoxigenic Bacteroidetes (9)	

In conclusion, based on previous studies, we have summarized a set of potential core microbiota markers for CRC, including cancer-promoting microbiota: Fusobacterium nucleatum, Parvimonas micra, Porphyromonas asaccharolytica, Bacteroides fragilis, Streptococcus gallolyticus and Cancer-inhibiting flora: Clostridium, Roseburia. These microflora may become future diagnostic biomarkers and therapeutic targets.

### 2.2 Mechanisms of microbiota for CRC progression

**Figure 1:**
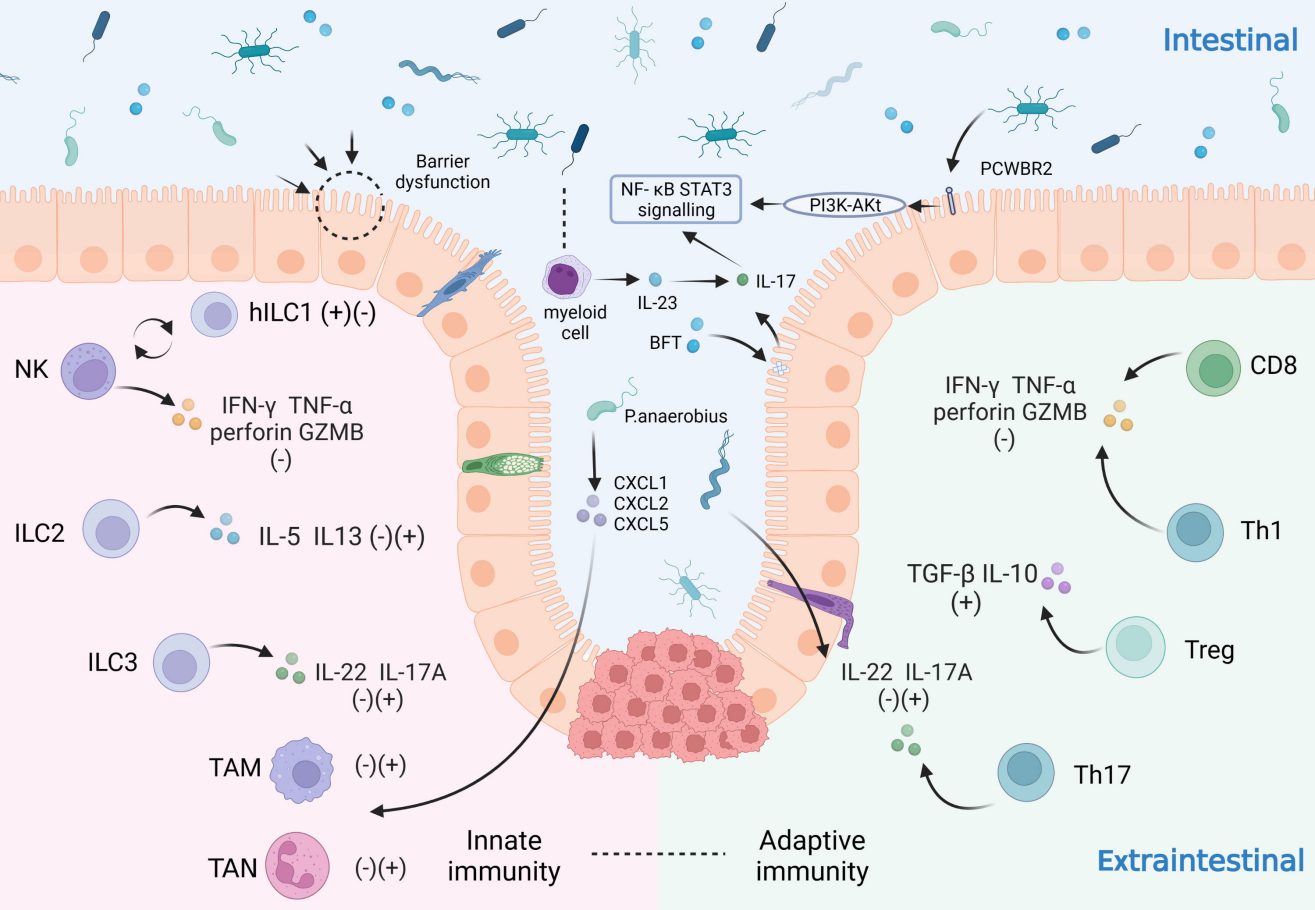
Influence of intestinal microbiota on the immune system in CRC patients. The immune system plays an important role in the development of CRC by virtue of its tumor-promoting and tumor-suppressing effects. The immune system can be categorized as the innate immune system and the adaptive immune system. In the innate immune system: NK cells secrete IFN-γ, TNF-α, perforin and GZMB to suppress tumors and can be converted to hILC1 under certain conditions; ILC2 secretes IL-5 and IL-3; ILC3 secretes IL-22 and IL-17A; tumor-associated neutrophils (TAN) and macrophages (TAM) also play a significant role; In the adaptive immune system: CD8+ T cells and Th1 cells secrete IFN-γ, TNF-α, perforin and GZMB; Treg cells secrete TGF-β and IL-10; Th17 cells secrete IL-22 and IL-17A. The innate and the adaptive immunity reinforce each other and play a pro-tumor or anti-tumor role together. The gut microbiota can influence on CRC cells through various mechanisms. For example: microbiota disrupt the gut barrier; some microbiota increase pro-inflammatory responses through PCWBR2 or activation of the NF-κB/STAT3 signaling pathway with lineage cells; others can act on domain CRC cells by regulating TAN, TAM through CXCL1/2/5.

At homeostasis, interactions between host cells and microbiota facilitate important symbiotic functions and maintain the structural integrity of the gut. However, this symbiotic relationship can become maladapted in CRC, including deterioration of the epithelial cell barrier when microbial invasion triggers inflammation, disrupts the tumor immune microenvironment and generates pro-oncogenic metabolites and bacterial toxins. The following section will underline the mechanisms of intestinal flora in relation to precancerous lesions and clinical transformation of CRC (**Figure 1**).

#### 2.2.1 Pro-inflammatory and immunomodulatory effects

Chronic inflammation is an important intrinsic factor that promotes carcinogenesis by inducing DNA damage, producing reactive oxygen and nitrogen species, regulating intestinal epithelial cell (IEC) polarization and the tumor microenvironment, activating transcriptional programs such as nuclear factor kappa-light-chain-enhancer of activated B cells (NF-κB) and STAT3 in IEC, and impeding anti-tumor immunity (17). Depending on IL-17 and the CEC IL-17R, enterotoxigenic Bacteroides fragilis (ETBF) colonization produces Bacteroides fragilis toxin to trigger strong selective distal colon NF-κB activation. The phenomenon reveals a STAT3- and Th17-dependent pathway of increased colonic tumor formation (18). Long XH et al. discovered in their constructed P. anaerobius-treated ApcMin/+ mice that P. anaerobius has a surface protein called putative cell wall binding repeat 2 (PCWBR2) which can bind α2/β1 integrin and activate the PI3K-Akt pathway in CRC tumor cells under the action of phospho-focal adhesion kinase. NF-κB in turn increased cytokine levels, such as IL-10 and IFN-γ, to stimulate the pro-inflammatory response (19). CRC patients have the higher abundance of Parvimonas micra compared with the healthy population. Colonization of P. micra upregulates genes involved in cell proliferation, stemness, angiogenesis and invasiveness/metastasis and enhances Th17 cell infiltration and Th17 secretion of cytokines (IL-17, IL-22 and IL-23) to promote CRC formation in mice (20).

#### 2.2.2 Metabolites

The role of gut microbota metabolites on CRC is a double-edged sword. Multi-kingdom microbiota analyses found CRC patients have 26 additional metabolic pathways versus healthy people, including pathways involved in carbohydrate metabolism (e.g. butyrate, ascorbate and aldehyde metabolism) and D-arginine and D-ornithine metabolism; 23 reduced pathways including branched-chain amino acid (valine, leucine and isoleucine) and lipid metabolism (e.g. phospholipase D) pathways (4). Naoki Sugimura et al. found that L. gallinarum can produce metabolites such as L-tryptophan that induce apoptosis in CRC cells (21). β-Galactosidase is a key protein. By secreting it, Streptococcus thermophiles can promote CRC cell apoptosis and mediate the anticancer effects of Streptococcus thermophiles by disrupting energy homeostasis, activating oxidative phosphorylation and downregulating Hippo pathway kinases with galactose production. Meanwhile, β-Galactosidase also increases the intestinal abundance of known probiotics such as Bifidobacterium and Lactobacillus (22). Specific intestinal bacteria, such as E. faecalis, E. roseus, Bifidobacterium, E. fungalis, and Lactobacillus, could ferment dietary fiber into short-chain fatty acids (SCFA), which are enteroprotective and negatively associated with colorectal cancer. SCFA, including butyrate, propionate and acetate, prevent CRC through mechanisms like modulation of intestinal inflammation, the immune system and so on (23). In addition to inhibiting CRC, some metabolites produced by the gut microbiota also promote CRC. M. morganii can produce indolimine to induce increased intestinal permeability and may lead to abnormal DNA replication and abnormal IEC proliferation *in vivo* to exacerbate the CRC burden (24).

#### 2.2.3 Bacterial toxins

Relying on METTL14-mediated N6-methyladenosine methylation, ETBF could downregulate miR-149-3p, which promotes proliferation of CRC cells (25). E. coli-produced colibactin can cause DNA damage and colibactin-producing E. coli (CoPEC) promotes the development of CRC in the mouse model with chronic inflammation induced by dextran sodium sulfate (DSS) and further enhances the pro-tumorigenic effect in the mouse model with IEC autophagy deficiency (26).

### 2.3 Therapeutic interventions for microbiota

Traditional strategies related to the prevention and treatment of CRC include probiotics, prebiotics, high-fiber dietary therapy, and fecal microbiota transplantation (FMT), which have been comprehensively reviewed (27). Still, these approaches are limited by their own drawbacks (e.g. low selectivity and specificity for specific sites of action and specific bacterial flora as well as safety issues in clinical translation). More useful therapeutic options targeting the intestinal flora are still expected (28). The above summary of the macrogenomic landscape of the CRC microbiota and the action mechanisms of the associated gut microbiota in CRC is valuable for the ensuing discussion of potential targets and clinical strategies for the diagnosis, treatment and prevention of CRC.

Targeting the ETBF/miR-149-3p pathway presents a promising approach to treat patients with intestinal inflammation and CRC with a high amount of ETBF. IEC autophagy inhibits CoPEC from inducing CRC occurrence in ApcMin/+ mice model, suggesting that targeted induction of autophagy may be a promising strategy to inhibit the pro-tumorigenic effects of bacteria, which could be achieved by immunotherapy and radiotherapy (29). Cai F et al. suggested that polyunsaturated fatty acids (PUFAs) have the potential to sensitize HT29 cells, possibly due to an increase in intracellular lipid peroxidation products (30). Short-chain fatty acids directed modulation in human and mouse CRC models enhances response to chemotherapy and immunotherapy, and targeting SCFAs and PUFAs for abundance reconstruction may be a new approach to managing CRC (31).

So, targeting gut microbiota holds the promise of achieving precise microbial regulation, satisfactory therapeutic outcomes, minimizing the possibility of adverse reactions, identifying effective primary prevention strategies and further reducing CRC risk.

## 3 Strategies for gut microbiota to increase the efficiency of CRC immunotherapy

The intestine is the largest immune organ of the body. The unique intestinal immune system is the basis of the body’s anti-tumor response; conversely, an imbalance in the intestinal immune system will facilitate the development of tumor. Meaningfully, the microbiota and the host have a mutually beneficial symbiosis. There is a close regulatory relationship between the microbiota and the host’s immune system. In short, the microbiota can modulate both innate and adaptive immune functions in the body, thereby influencing oncogenesis and anti-tumor immune function.

### 3.1 The role of microbiota on the immune system

Innate immunity is a natural defense that produced in order to adapt to the environment. The main innate immune cells in the intestine are neutrophils, macrophages and innate lymphocytes (ILCs). TAN can be classified into an anti-tumorigenic “N1” phenotype and a pro-tumorigenic “N2” phenotype (32). The “N1” phenotype increases cytotoxicity through producing TNF-α, reactive oxygen species (ROS), etc. (32). Conversely, the “N2” phenotype promotes tumor development through the expression of arginase and various chemokines (33). The phenotype of TAN depends on the tumor microenvironment (TME). For example, TGF-β induces the “N2” phenotype, while the “N1” phenotype is induced by IFN-β (32). TAM can be classified as “M1”, which exerts pro-inflammatory effects, and “M2”, which exerts anti-inflammatory and tumor-promoting effects. It has been suggested that a high pan-macrophage density at the margins of tumor infiltration has a positive impact on the prognosis of CRC patients, while the opposite result is observed in the center (34). As the main intestinal ILC cells, ILC1 and NK cells regulate the different steps of CRC development. It has been suggested that NK cell can transdifferentiate to a less cytotoxic ILC1-like phenotype in the presence of TGF-β, which is present in the TME of CRC (35). It has also been shown that high ILC1 levels may predict poor cancer prognosis (36).

Gut microbiota can regulate the innate immune system in various ways. Toll-like receptors (TLRs) bind to ligands to coordinate early host resistance to infection through signaling pathways such as NF-κB and mitogen-activated protein kinases (MAPK) (37). Specifically, TLR2 can form heterodimers with TLR1 and TLR6, which initiate MyD-88-dependent signaling pathways regulating cytokine transcription. Lactobacillus fermentum can identify TLR2/TLR1 and TLR2/TLR6 signaling (37). In intestinal injury associated with inflammatory bowel disease, TLR4 increases its sensitivity to LPS through the release of IFN-γ and TNF-α (38). Lactobacillus and Bifidobacterium suppress animal enteritis by reducing TNF-α by TLR4 (39). It has been shown that the promotion of IFN-γ release *via* TLR9 by S. amoebae may contribute to cytokine imbalance in UC (40). Abnormal TLR signaling activation in immune cells may also lead to the release of pro-inflammatory cytokines, resulting in acute or chronic intestinal inflammation (41).Additionally, TLRs, activated by microbiota, can also start adaptive immune responses.

Adaptive immunity is produced by lymphocytes through contacting with antigenic material and is specific and memory-based. Adaptive immunity in the gut occurs mainly in the lymphoid tissues associated with the gut (e.g. Pai’s node, mesenteric lymph nodes), while T and B cells in the lamina propria also play an important role.

B cells can identify CRC antigens and produce specific antibodies in cooperation with helper T cells, thereby impeding CRC development and progression. CD8+ T cells are the immune cells that specifically target tumor cells. A study showed that IL-18 was highly expressed in 72% of tumor cells in CRC and can act as a trigger to prompt a series of immune responses from CD8+ T cells (42). Additionally, CD8+CD279+ cells could be a potential biomarker for predicting postoperative prognosis in CRC patients (43). CD4+ T cells can be divided into Th1 cells and regulatory T cells (Treg). Th1 cells are functionally similar to CD8+ T cells, while Treg cells exert immunosuppressive functions through IL-10, TGF-β. A study has shown that the density of combination of CD4+ and FOXP3+ cell is a precise prognostic marker. Furthermore, only one type, such as CD4+ or FOXP3+ cells, may be sufficient for a suitable TME to prevent recurrence (44). Th17 cells and IL-17 can influence the process of tumor through various mechanisms, including immune infiltration, promotion of cancer cell invasion and metastasis, etc. A study identified related genes (KRT23, ULBP2, ASRGL1, SERPINA1, SCIN, SLC28A2) that may affect the immune infiltration of Th17 cells in COAD patients and suggested that the effect of these genes on Th17 cells may be responsible for their dual product (45).

Gut microbiota can modulate adaptive immune function by stimulating the differentiation of T cell in the gut and by regulating T cell antigen recognition and tumor killing functions. Studies have shown that antigenic peptides derived from bacteria such as Akkermansia muciniphila can induce differentiation of Treg cells in the colon and improve intestinal inflammation (46). Short-chain fatty acids (SCFA) can increase Treg cells differentiation (47, 48), while SFB and S. fragilis induce differentiation of Th17 cells (46). In summary, as an important component of the CRC tumor microenvironment, the immune system interacts with the gut microbiota to control inflammation and anti-tumor immunity.

### 3.2 Potential for microbiota to improve ICB

ICB refers to suppressing tumor immune evasion and enhancing anti-tumor immunity by inhibiting the interaction between IC and tumor cells through IC inhibitors (ICI). Currently the immune checkpoint molecules PD-1 and CTLA-4 have been recognized as important targets for immunotherapy of CRC.

PD-1, a protein in the CD28 family on the surface of activated immune cells, binds to PD-L1 on the surface of tumor cells. It can block the PI3K pathway and thus inhibit T cell activation. CTLA-4 promotes tumor immune evasion by competitively binding to B7 ligands and inhibiting the function of Treg cells. At present, two PD-1 blocking antibodies, pembrolizumab and nivolumab, and the CTLA-4 receptor blocking antibody ipilimumab are approved by FDA for the treatment of dMMR/MSI-H CRC (49). Although ICBs offer a new direction for the treatment of cancers, the need to improve ICB efficacy and reduce immune-related adverse events (irAEs) continues to be a hot topic of research.

Recent studies have shown an association between higher levels of tumor mutational load (TMB) and improved survival for patients treated with ICB (50). Compared to the pMMR/MSS phenotype, The dMMR/MSI-H phenotype generally shows higher levels of TMB and immune cell infiltration (51). It has been shown that immunotherapy is effective in some cases of dMMR/MSI-H CRC, while many patients in the pMMR/MSS phenotype show resistance (52). For ICB, although dozens of clinical trials have demonstrated the effectiveness of ICB and it has received several FDA approvals, its response has been limited to patients with dMMR/MSI-H CRC (53). A recent meta-analysis showed that the MSS-H/dMMR subgroup had a higher ORR compared to the pMMR/MSS subgroup when treated with anti-PD-1/PD-L1 antibodies (54). However, the dMMR/MSI phenotype accounts for approximately 15-18% of CRC patients and 5% of metastatic CRC (mCRC) patients (55). This means that most CRC patients are the pMMR/MSS phenotype, and they often don’t have satisfactory results after receiving ICB. Therefore, it is important to improve the efficacy of dMMR/MSI-H phenotype immunotherapy and explore new immunotherapies that benefit pMMR-MSI-L.

ICB may alter the composition of the patient’s gut microbiota. A study analyzing patients with gastrointestinal cancers treated with anti-PD-1/PD-L1 therapy showed elevated relative abundance of Prevotella and Bacteroides in responders (56). In a mouse model of CRC, the ileal microbiome controlled the efficacy of PD-1 blockers in CRC (57). In addition, nonprotective E. faecalis can expresses sufficient SagA to enhance the anti-tumor effects of PD-L1 in mice (58). Cell lysates of Lactobacillus acidophilus combined with CTLA-4-blocking antibodies can enhance antitumor immunity in a mouse CRC model (59). Notably, because the composition of good or harmful microbiota may vary depending on the location of the tumor, further research is still needed in the field of immunotherapy for CRC. In addition, some studies have shown that irAEs are associated with reduced diversity and altered composition of the gut microbiota. A clinical study analyzed the microbiota of patients with advanced non-small cell lung cancer treated with PD-1 antibodies. There were significant differences in microbiota composition in patients with diarrhea compared to non-diarrhea patients, as evidenced by non-diarrhea patients exhibiting higher abundance of Bacillus mimicus and lower abundance of thick-walled bacteria (60). This suggests that gut microbiota interventions may not only improve the efficacy of ICB, but may also be a starting point for the treatment and prevention of irAEs.

There are some perspectives on the mechanisms. On the one hand, the gut microbiota improves ICB efficacy through innate immunity. It has been demonstrated that microbiota can induce intra-tumoral monocyte production of IFN-1 to modulate TAM, which would ultimately improve ICB efficacy (61). It has also been found that an increase in bifidobacteria within the tumor will enhance NK cell function and thus enhance the therapeutic effect of PD-1 blockers (62). On the other hand, microbiota may also improve ICB efficacy through adaptive immunity. One study isolated a bacterial community of 11 types of bacteria from healthy human donor faeces. The bacterial community can induce CD8+ T cells in the gut and enhance the therapeutic efficacy of ICI in a tumor model (63). Other mouse models have also demonstrated that Lactobacillus acidophilus can improve anti-CTLA4 therapeutic efficacy in CRC by reducing Treg cells and “M2” TAM and by increasing CD8+ T cells (59). A phase I clinical trial found a relative increase in the abundance of enterococci in refractory metastatic melanoma following the use of PD-1 blockers and FMT, which led to intra-tumoral CD8+ T-cell infiltration and ultimately better tumor killing (64).

In addition to modulating the innate and adaptive immune system, microbiota can also enhance the immunogenicity of tumor cells to improve ICB efficacy. On the one hand, the gut microbiota metabolite inosine can directly enhance tumor intrinsic immunogenicity through UBA6 (65). On the other hand, microbiota can provide tumor cross-antigens and thus indirectly increase the immunogenicity of tumor cells (66). It is worth mentioning that metabolites of the gut microflora, such as inosine, short-chain fatty acids (SCFAs) (67, 68), arachidonic acid (66), bile acids and tryptophan are considered to be effective targets for improving the efficacy of ICB.

### 3.3 Strategies for improving ICB with gut microbiota

#### 3.3.1 Fecal microbiota transplantation (FMT)

We know that patients with good gut microbiota status can improve the TME through microbiota, thereby enhancing the efficacy of ICB (69). FMT, which involves transplanting faecal microbes from patients who have responded to ICB into non-responders, holds promise for improving the efficacy of ICB and reducing irAEs. Previously, mice from the Jackson Laboratory (JAX) showed increased efficacy about anti-PD-1 immunotherapy, suggesting that the gut microbiota influence the efficacy of anti-PD-L1 therapy (70). A recent study found a stronger anti-tumor effect of PD-1 blockers in the faecal microbiome of cancer patients transplanted with a response to ICB compared to those transplanted with no response to ICB in mice that were germ-free or on antibiotics (71). Based on that, a number of clinical studies aiming at assessing the therapeutic potential of FMT-enhanced ICI are underway, mainly involving melanoma, prostate cancer, gastrointestinal tract cancer and lung cancer (72). However, there are fewer clinical studies on CRC.

In addition, one study demonstrated in CRC mice that FMT exerts an anti-inflammatory function by restoring the ratio and diversity of gut microbiota, which also suggests that “ FMT + ICI” treatment may not only improve the efficacy of ICB treatment but may also reduce irAEs (73). However, before FMT is performed, donors are screened regularly to limit the spread of microorganisms that may cause infection. The safety of FMT also needs to be observed with long-term follow-up.

#### 3.3.2 Probiotics

Prebiotics, probiotics and commensal bacteria have been studied in relation to anticancer treatment strategies such as chemotherapy and radiotherapy, but less research has been conducted on ICB. Excitingly, there is still considerable evidences that this therapy is beneficial in improving ICB efficacy and reducing immune-related adverse events.

A recent study, using a combination of anti-ePD-L1 and prebiotic Bilberry Anthocyanin to treat CRC in mice, suggested that prebiotics may improve ICB efficacy by restoring microflora diversity (74). Other studies in mice have also shown that probiotics such as Bifidobacterium and Mucorophilus in combination with ICB can enhance therapeutic efficacy (70). Similarly, administration of Lactobacillus rhamnosus GG enhances anti-PD-1 therapeutic efficacy by promoting CD8+ T cell function (75). Interestingly, another mouse model showed that administration of a probiotic (Bifidobacterium longum or Lactobacillus rhamnosus GG) reduced the frequency of IFN-γ(+)CD8+ T cells exhibiting an unfavourable antitumor response. Therefore, the mechanism of probiotics in ICB treatment still needs further research (76). In addition, there are also studies that show probiotics are associated with irAEs. One study found that administration of vancomycin may enhance the immunopathological response to ICB, which was associated with depletion of Lactobacillus. Further studies found that administration of the probiotic Lactobacillus reuteri completely eliminated ICB toxicity (77). It is important to note that although there are relevant data observed in mouse models, clinical evidence must be available to support the use of probiotics before they can be encouraged.

## 4 Microbiota and CRC radiotherapy

Currently, most early CRC can be cured by surgical resection, but advanced CRC are difficult to eliminate completely by surgery and require multimodal treatment including chemotherapy and radiotherapy as well as surgery. Especially, due to the proximity of the rectum to the pelvic organs, the absence of plasma serosa around the rectum and the technical difficulties in achieving wide surgical margins, radiotherapy has been established as the main treatment option for patients with advanced colorectal cancer in addition to surgery.

It is noteworthy that the results of a growing number of studies prove that the final outcome of radiotherapy is closely related to biological factors (78). There is a two-way interaction between intestinal microbiota and radiotherapy. On the one hand, intestinal microbiota affects the antitumor clinical efficacy of radiotherapy, on the other hand, ionizing radiation changes the composition and function of intestinal microbiota, which in turn leads to the development of radiation enteropathy (79). Intestinal flora is of significant interest for its use in radiotherapy, as a protective agent and a biomarker in radiation exposure.

### 4.1 Mechanisms of microbiota in CRC radiotherapy

Next we focus on the two main mechanisms of ionizing radiation-induced dysbiosis of the intestinal flora. First, radiation can damage IEC, resulting in impaired intestinal barrier function, allowing bacteria to move deeper into the body and promoting the entry of bacterial metabolites into the bloodstream, which promotes inflammation (80). Second, radiation exposure can cause the formation of ROS, which are chemically active due to their unpaired electrons and can damage the DNA and other cellular structures of the intestinal microbiota, thereby triggering changes in the bacterial flora (81). In addition, the various microorganisms that make up the intestinal microbiota have different intrinsic radio-sensitivities (82), so radiation exposure can alter the composition and relative proportions of the microbiota.

El Alam et al. (83) found that in patients treated with pelvic chemoradiotherapy, gut microbiome composition and relative abundance continually decreased in the overall. Although the diversity of the population’s gut microbiota tended to return to baseline levels during the 12-week follow-up period, there are still significant changes in structure and composition, the most notable of which was the increase in the number of Bacteroidetes. Pooled results from other studies suggest that the inflammatory environment produced by radiotherapy leads to increased abundance of pathogenic pro-inflammatory bacteria (e.g. S. wadsworthensis and S. parvirubra) and mimics; decreased abundance of anti-inflammatory bacteria (e.g. E. faecalis and Prevotella histicola) and Phylum Firmicutes including Lachnospira pectinoschiza, Roseburia intestinalis, etc (84, 85). By sequencing the 16s rRNA gene in the mouse model, significant changes in gut microbial composition could be observed in the radiation background. Moreover, changes in the flora can lead to changes in their metabolites, such as lactic acid, which has a protective effect on the body (86). The foregoing studies imply the homeostasis dysregulation of intestinal flora and its metabolites after radiotherapy provides a potential target for improving the prognosis of CRC patients treated with radiotherapy.

Microbial colony metabolites may affect radiosensitization or radioresistance. ETBF has been proven to be enriched in CRC patients as a potential cancer-promoting colony, and it was found that Bacteroides fragilis toxin (BFT) and IL-17 produced by ETBF after colonization synergistically activate the STAT3 signaling pathway in IC (18). Park SY, et al. found that, JAK2/STAT3/CCND2 axis is a key mediator of radioresistance (87). Thus, we speculate that ETBF may enhance the radioresistance of CRC by generating BFT.

Based on these links, microbiota may be a modulating factor for ameliorating radiation adverse effects and radiation toxicity. Baseline gut microbiota diversity is a predictor of the extent of change in gut flora diversity during CRT. El Alam et al. (83) unexpectedly discovered that patients with high intestinal flora diversity at baseline had a greater decline in intestinal flora diversity from the start to the fifth week of CRT than patients with low intestinal flora abundance at baseline. This finding suggests that the optimal target group for CRT intervention may actually be patients with high baseline gut richness and diversity rather than the low. Interestingly, in the same year there was new evidence that patients with abundant microbial diversity had increased activation of CD4+ lymphocytes infiltrating cervical tumors as well as CD4 cell subpopulation expressing ki67 and CD69+ during radiotherapy (88). Considering that the combination of radiotherapy and immunotherapy can strengthen the anti-tumor immune microenvironment to the maximum extent and it has been demonstrated that intestinal flora can promote the efficacy of immunotherapy (70). We hypothesize that high abundance of gut microbiota may enhance the sensitivity of tumor cells to radiotherapy through immunomodulation. Given the similarities between cervical and colorectal cancer in terms of irradiation sites and modalities, we suspect that a similar effect might be achieved in radiotherapy for CRC.

### 4.2 Strategies associated with targeted colonies to improve the efficacy of radiotherapy

Results from experiments using an orthotopic syngeneic murine model of breast cancer treatment with focal irradiation as a mouse model indicated that treatment with an antibiotic (Abx) cocktail of ampicillin, imipenem, and cilastatin prior to radiotherapy reduced therapeutic efficacy, and a similar reduction was observed in a previous melanoma model. In contrast, the combination of radiotherapy and the fungal antibiotic enhances the clinical efficacy of RT and improves the immunosuppressive tumor microenvironment by increasing granzyme B-producing CD8 T cells (89). The results draw our attention to the potential of manipulating intestinal fungal flora through antibiotics to enhance antitumor immune responses to radiotherapy. Vancomycin, a glycopeptide antibiotic active against gram-positive bacteria, alters the composition of the intestinal microbiota when combined with radiotherapy in a preclinical model, and leads to increased antigen presentation of CD11c dendritic cells in tumor-draining lymph nodes of radiotherapy mice, which enhances the antitumor effect (90). Recently, the results of Yang K et al. showed that the vancomycin effect was abolished by butyrate, so that butyrate-producing bacteria, such as Lactobacillariophyceae and Rumenococcaceae, may be new therapeutic targets (91). At the meantime, we should also notice that inappropriate antibiotic use in ICI-treated patients may weaken treatment outcomes due to antibiotic-induced ecological dysregulation (92). Therefore, when selecting antibiotics, especially broad-spectrum antibiotics, it is critical to consider the potential risks of antibiotic therapy for cancer patients, including potential adverse effects on treatment efficacy and toxicity.

### 4.3 Radiation enteropathy and microbiota

Radiation changes microbiota and produces radiation toxicity. Pelvic irradiation is one of the methods of treatment for CRC. Although RT has a positive killing effect on cancer cells, radiation damage to normal organs and tissues inevitably occurs during radiotherapy, especially for one of the most radiosensitive organs, the intestine. The intestinal damage caused by radiation is known as radiation enteropathy (RE), and radiological diarrhea (RID) is most common symptom. Frequent or persistent diarrhea has been reported in 51.9% of women treated with standard radiotherapy and 33.7% of women treated with intensity-modulated radiotherapy (93), which greatly reduces the patient’s quality of life after surgery and is also likely to affect the efficacy of radiotherapy through delayed radiotherapy.

As RE has received increasing attention, the mechanisms by which RE occurs are being studied more and more clearly. There are considerable evidences that gut microbiota play an important role in the development of RE during pelvic irradiation and after treatment.

What is clear is that there are differences in the gut microbiota between those who develop diarrhea and those who do not in patients receiving radiotherapy. In experimental animals, germ-free mice are thought to exhibit less radiotoxicity than conventionally reared mice, suggesting that gut microbiota may influence radiation-induced intestinal toxicity (94). Similarly, in a mouse model of acute radiation-induced intestinal injury (RIII), acute RIII was found to occur with reduced diversity of gut microbiota, reduced abundance of beneficial bacteria and increased abundance of pathogens. Therefore, it is likely that the gut microbiota are potential biomarkers for the critical phase of RIII (95). In rectal cancer patients, Bifidobacterium, Clostridium and Synechococcus are enriched in patients without Diarrhea or Mild Diarrhea (96). In cervical cancer patients treated with pelvic radiotherapy, RE patients had significantly lower α-diversity but increased β-diversity of gut microbiota, with relatively high abundance of Aspergillus and Gammaproteobacteria and lower abundance of Aspergillus. Interestingly, Coprococc was found to be significantly enriched in patients who subsequently developed RE before receiving radiotherapy and had a graded associated microbial profile, suggesting that Gut microbial dysbiosis may be a potential biomarker for human RE (97). A recent systematic evaluation showed that in patients undergoing pelvic radiotherapy, the levels of thick-walled bacteria, Aspergillus and Actinobacteria, were higher in the gut of patients with diarrhea compared to those without diarrhea, while most posterior and anaphylactic bacteria were lower. And at the genus or class level, patients with diarrhea had the presence of Sutrobacter, Fine Golden Bacteria, Peptococcaceae (Clostridium), Prevotella 9, Faecalis, Desulfovibrio, Anaplasma, Verrobacter, Dictyostelium and Bacteroides, while intestinal dominant bacteria (e.g. Clostridium, Anaplasma, Brautia, Ruminococcaceae UCG-003, Faecalis Bacillus oscillatus, Prevotella and Roscoe) were reduced (98). In conclusion, there is an association between RE and dysbiosis of the gut microflora, more consistently: reduced diversity and abundance of microflora, increased abundance of pathogenic bacteria (e.g. Aspergillus, Clostridium) and reduced beneficial bacteria (E. faecalis, Bifidobacterium, etc.) (98).

#### 4.3.1 Possible mechanisms between microbiota and RE

The relationship between microbiota and RE may be related to mechanisms such as inflammation, disruption of the epithelial barrier and intestinal permeability, and the release of immune molecules. One study proposes that, the gut microbiota are dysregulated following irradiation. This is likely to directly induce intestinal barrier dysfunction and inflammatory responses. The trial specifically cultured normal colonic epithelial cells with faecal bacteria from patients with severe RE and found that this increased intestinal permeability and stimulated cytokine and NF-κB activation (97). In another experiment, researchers found that irradiated microbiota stimulated increased secretion of IL-1β, which exacerbated radiation-induced intestinal tissue injury. Tissue injury improved after IL-1 was blocked, suggesting that IL-1β is involved in at least part of the microbiota-mediated radiation-induced intestinal injury (84). In addition, the metabolism of the gut microbiota is also disturbed to some extent after irradiation, especially lipid metabolism. It is well known that the lipid bilayer is the basis of the intestinal epithelial barrier and therefore disorders of lipid metabolism may also be an important factor in the development of RE.

However, studies on how the gut microbiota are involved in radiation-induced intestinal damage are still scarce, and it is necessary to clarify the mechanisms involved before applying microbial agents to improve RE. Next we will focus on strategies for reducing radiotoxicity through microbiota.

#### 4.3.2 Strategies for reducing radiotoxicity through microbiota

**Radiotherapy + FMT, probiotics, prebiotics, diet**: Based on the potential role of gut microbiota in reducing radiotoxicity, several studies have begun to explore whether adverse effects caused by pelvic irradiation can be minimised by gut microbiota, with FMT and probiotics receiving the most attention.

An experimental study demonstrated that FMT attenuated acute radiation syndrome (ARS), and further studies found that FMT increased indole 3-propionic acid (IPA) levels in the faecal microbiota of irradiated mice, and that IPA ameliorated gastrointestinal toxicity after total abdominal irradiation without accelerating tumor growth (99). In a recent case report, investigators followed a patient who developed chronic hemorrhagic radiation proctitis after radiotherapy for cervical cancer. Significantly, after four courses of FMT treatment, the patient experienced relief of symptoms such as blood in the stool, abdominal pain and diarrhea, and significant changes in intestinal bacterial tests (100). In addition, a clinical study has shown that FMT can safely and effectively improve bowel function over time in CRE patients with chronic radiation enteritis (101). This further suggests the possibility of FMT in reducing the gastrointestinal toxicity of radiotherapy. It is worth noting that clinical studies on FMT and radiation enteritis induced by CRC radiotherapy, as well as studies on the mechanisms involved, are scarce, and the intestinal effects of FMT on the recipient are complex and unpredictable. Therefore, a large number of studies are still needed to demonstrate the feasibility and safety of FMT before it can be truly used in the clinic.

Probiotics such as Lactobacillus and Bifidobacterium have been shown to prevent gastrointestinal toxic reactions such as colitis and diarrhea. As microorganisms that play a beneficial role in cancer prevention and treatment, probiotics are thought to reduce the translocation of harmful bacteria as well as protect intestinal immune barrier function.

One experiment showed that the probiotic Lactobacillus rhamnosus GG has a radioprotective effect on the mouse intestine, possibly through the release of lipoteichoic acid, macrophage activation and the migration of mesenchymal stem cells (102). Similarly, in the clinical setting, one study found that Lactobacillus rhamnosus GG reversed intestinal ecological dysregulation and diarrhea during cancer treatment (103). Interestingly, a meta-analysis noted that the widespread use of probiotic interventions for diarrhea secondary to cancer therapy did not show positive results (104). This suggests that research efforts should be focused on specific gastrointestinal toxicity as well as unique probiotic pairings. There is still a paucity of objective clinical evidence on the beneficial effects of probiotics on radiation gastrointestinal toxicity in CRC, and a lack of corresponding studies on how probiotics are formulated, administered and absorbed.

**FLASH-Radiotherapy (FLASH RT)**: There are growing evidences that radiation can disrupt the gut microbiome and cause dysbiosis of the gut ecology, which in turn affects the effectiveness of radiotherapy as well as increasing radiotoxicity (79, 83). Significantly, recent studies have shown that FLASH irradiation can reduce changes in the gut microbiome compared to clinically conventional radiotherapy (CONV) (105). FLASH-RT is a new type of radiotherapy that will deliver a dose at an ultra-high dose rate (≥ 40 Gy/s) compared to CONV, which is thousands of times higher than CONV. FLASH-RT can significantly protect healthy tissue from radiation damage without altering tumor killing function. In 2019, the first patient with T cell cutaneous lymphoma underwent FLASH-RT and showed good results for both normal skin and tumor, demonstrating the clinical feasibility and safety of FLASH-RT (106).

A very important feature of FLASH-RT is the short exposure time, which may reduce the proportion of immune cells killed, thus allowing the immune system to exert more robust anti-tumor immunity as well as repairing normal tissue damage (107). On the immune cell side, it has been demonstrated that FLASH-RT promotes better recruitment of CD3+ T cells to the tumor core compared to CONVi, as well as higher levels of cytotoxic CD8α+ T cells in the TME (108). In terms of immune molecules, FLASH-RT can reduce TGF-β expression (109, 110), and low levels of TGF-β improve anti-tumor immune responses and inhibit Treg cells differentiation. In conclusion, FLASH-RT can improve antitumor immunity through immune molecules, but more studies are needed to confirm the role of the immune system in the FLASH-RT response.

Since the gut microbiota and the gut immune system are interdependent and influence each other, we speculate that it is likely that the gut microbiota also play a role when receiving FLASH-RT, which may eventually manifest itself through improved immune system function. It so happens that one study is consistent with our suspicions, and this study suggests that FLASH irradiation greatly reduces changes in the gut microflora compared to CONV irradiation (105). The ecological dysbiosis of the gut microflora is often an important manifestation of a dysregulated intestinal immune system, and a dysregulated immune system is a detrimental factor for both anti-tumor immunity and the protection of normal tissues.

However, further studies are still needed to confirm the role of the gut microbiota in modulating the effects of FLASH. It is worth mentioning that studies on FLASH and CRC are currently scarce and further studies are also still needed to confirm the feasibility and safety of FLASH in the treatment of CRC patients.

## 5 Potential association between microbiota and radioimmunotherapy for CRC

In addition to inducing DNA damage, local radiotherapy can also promote anti-tumor immunity. New insights in the field of cancer therapy suggest that radiotherapy carries out antitumor by enhancing immunogenicity, including increasing the sensitivity of cancer cells to killing by cytotoxic T cells (111), enhancing antigen processing and inducing the expression of unique radiation-associated peptides in cancer cells (112). Inducing irradiated cancer cells to release or express immunogenic molecules that enhance the anticancer immune response (113) and facilitating the regulation of TME for immune-mediated antitumor effects. CD8+ cytotoxic T lymphocytes (CTL) play an important role in these processes (114). Combination of radiotherapy with immunostimulatory anti-PD1 and anti-CD137 mAbs produces favorable effects on distant non-irradiated tumor lesions and the therapeutic activity is carried out by CD8 T cells (115).

cGAS is a kind of cytosolic dsDNA sensor, STING is a type of endoplasmic -reticulum-resident protein. When bound to dsDNA, the nucleotidyl transferase activity of cGAS is stimulated, triggering a signaling cascade reaction involving STING, which results in the production of type I IFN (116) to initiate the innate immune response and generate the subsequent adaptive anti-tumor immune response. DNA released from dying tumor cells may trigger IFN-α/β *via* STING, which in turn may act on both cross-triggered DC and CD8 T cells as necessary factors to favor CTL immune responses. Strategies aimed at local enhancement of IFN-α/β can make radiotherapy-induced tumor cell death more immunogenic (117). Thus, tumor cells killed by radiotherapy are immunogenic. cGAMP treatment and radiotherapy synergistically amplify the antitumor immune response, and the synergistic effect depends on the presence of STING in the host.

TAM are considered to be important immune cell components of the tumor microenvironment and are abundant myeloid cells in the stromal lumen of varied solid tumors (118). Radiotherapy activates the differentiation of M1 macrophages, promotes the influx of M1 macrophages into tumor cells and prevents the conversion to M2 type for the sake of ensuring the therapeutic effect. RT reprogrammed macrophages have a profound impact on tumor therapy. The conversion of the M2 to M1 phenotype promotes tumor therapy and acts as an implicit mediator of abscopal effects (119).

At the same time, however, it was found that RT-induced immunomodulatory effects are a double-edged sword. To some extent, radiotherapy also promotes immunosuppressive effects, as demonstrated by increased recruitment of MDSC, Treg and anti-inflammatory macrophages (M2 macrophages) (120). There is a balance between immunosuppression and immunostimulation. Without intervention, the function of CD8+ T cells is not sufficient to completely eradicate residual tumor cells under this balance, causing tumor recurrence and limiting the therapeutic effect of local radiotherapy. Many research results have demonstrated that the addition of immune checkpoint inhibitors can break this balance and optimize the superiority of CD8+ in anti-tumor (121).

**Figure 2:**
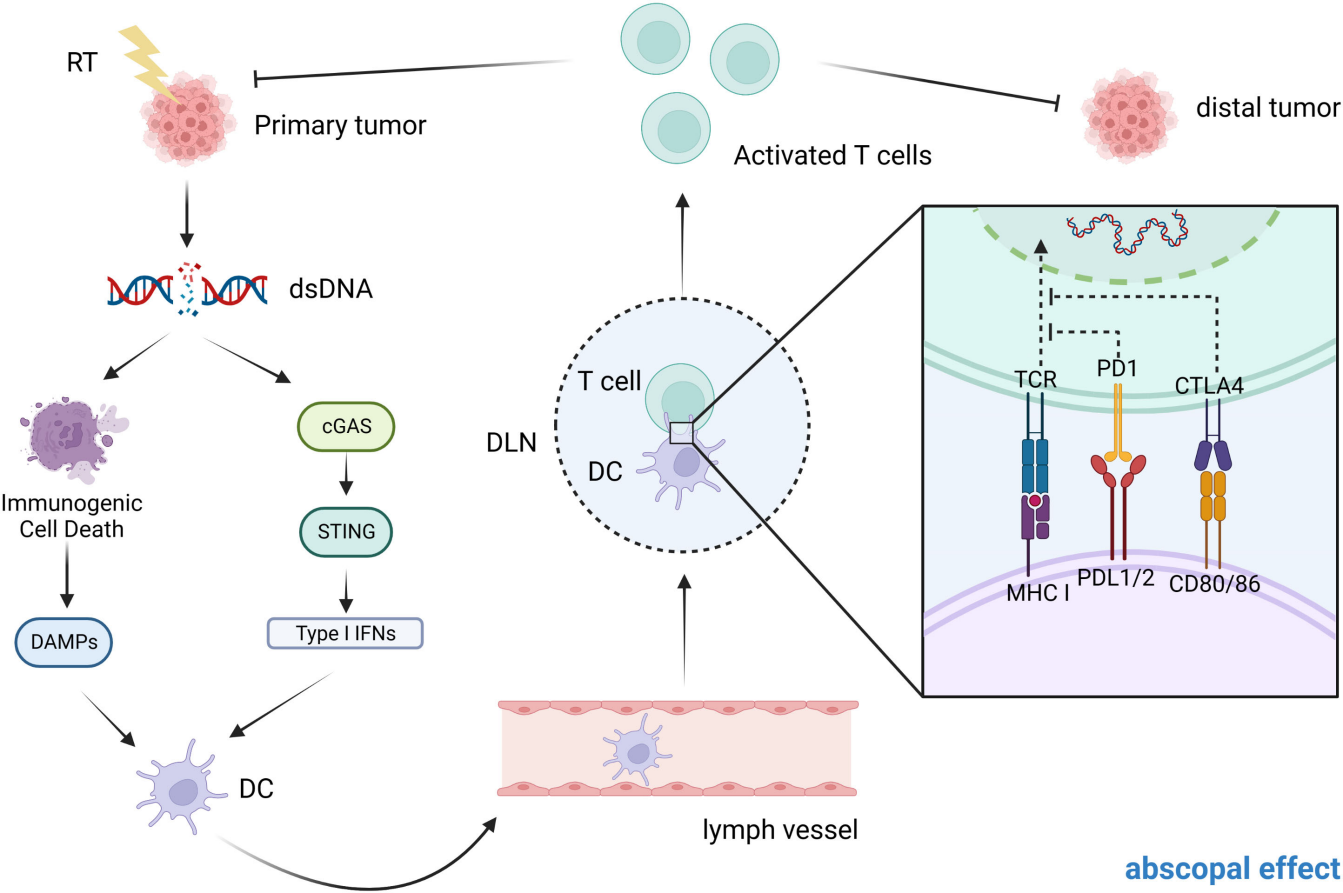
Possible mechanism of abscopal effect in CRC patients. After exposure to ionizing radiation, the primary tumor may undergo dsDNA breaks and this will cause a number of reactions. On the one hand, it can cause immunogenic cell death, which in turn releases DAMPs. On the other hand, dsDNA breaks activate the cGAS-STING signaling pathway, releasing type I IFN. Both of these results lead to the activation of DCs, which in turn activate CD8+ T cells (by presenting tumor antigens released from dying tumor cells) to mediate a specific anti-tumor immune response. In patients with metastatic tumors, when one tumor is irradiated, both of these modes can activate T cell activation and migration through the circulatory system to distant sites to induce the abscopal effect.

Immunotherapy also promotes the abscopal effects of radiotherapy. Several cases have confirmed that radiotherapy combined with anti-PD-1/PD-L1 is effective in controlling the development of tumors outside the radiation field. (**Figure 2**) Although it has been extensively studied, the specific mechanism of radiation-induced distant abscopal effect (RIAE) still needs further demonstration (114).

In CRC, although immunotherapy has shown some benefit in patients with the MSI-H/dMMR phenotype, the benefit is modest in the larger proportion of patients with MSS/pMMR phenotype. However, several recent studies have shown that radiotherapy combined with immunotherapy may improve the efficacy of immunotherapy as a viable and safe option for patients with MSS/pMMR phenotype. For example, a phase II clinical trial demonstrated that patients with MSS/pMMR rectal cancer treated with a combination of PD-1 inhibitors and nCRT demonstrated positive cCR rates and good tolerability (122). It is worth mentioning that the abscopal effect produced by radiotherapy combined with immunotherapy has also shown a through positive effect in the treatment of patients with colorectal cancer liver metastases (123).

Although positive and safe efficacy has been observed in a variety of solid tumors, clinical studies on radiotherapy-ablative combination therapy for CRC are still relatively few and the results of valuable studies are scarce, so further animal studies and clinical trials are needed to demonstrate the feasibility and safety of radiotherapy-ablative combination therapy for CRC patients, especially for MSS/pMMR phenotype who are not sensitive to immunotherapy and safety (**Table 2**).

**Table 2:** Current clinical trials on combination therapy in CRC patients.

Clinicaltrials. gov. identifier	Type of trial	Status	immunology	radiotherapy	Primary outcome/endpoint	Reference
NCT02888743	phase II	Active,not recruiting (2017/06- 2022/11)	Durvalumab Tremelimumab	radiotherapy	Overall response rate	(124)
NCT04124601	phase II	Recruiting 2020/06- 2023/05)	Ipilimumab Nivolumab	Chemoradiotherapy	adverse events	(125)
NCT05215379	Phase II phase III	Recruiting (2022/10- 2023/04)	xintilimab (injection)	neoadjuvant chemoradiation therapy	cCR	(122)
NCT04892498	phase II	Recruiting (2021/05- 2023/08)	PD-1 inhibitor	Hypofractionated radiotherapy	PFS	(126)
NCT04304209	Phase II phase III	Recruiting (2019/10- 2021/10)	Sintilimab	radiotherapy	pCR	(127)
NCT03503630	phase II	Active, not recruiting (2018/07- 2024/01)	COMPOUND 2055269	radiotherapy	pCR	(128)
NCT04109755	phase II	Recruiting (2020/06- 2022/06)	Pembrolizumab	SCRT	TRG	(128)
NCT03104439	phase II	Recruiting (2017/05- 2024/07)	Nivolumab Ipilimumab	radiotherapy	CR PR SD	(129)
NCT03101475	phase II	Completed (2018/11- 2022/02)	Durvalumab (MEDI4736) Tremelimumab	SBRT	iBOR	(129)
NCT02888743	phase II	Active, not recruiting (2017/06- 202212)	Durvalumab Tremelimumab	radiotherapy	Overall response rate	(129)
NCT02437071	phase II	Active, not recruiting (2015/04- 2023/04)	Pembrolizumab	radiotherapy	response rate	(129)

### 5.1 Advantages of CRC radioimmunotherapy

For CRC patients, surgical treatment is ineffective in treating distant metastases and requires artificial fistulas, which are risky to metastases and recurrence. There is a consensus that combined radiotherapy and immunotherapy can improve the chance of distant septal effects, and its clinical effect on eliminating metastatic tumors in patients is remarkable, which improves the recurrence rate and quality of survival. Preclinical studies have shown that after isolated radiotherapy, IFN-γ produced by CD8+ T cells mediates the upregulation of PD-L1 on tumor cells and induces local antitumor response. Radiotherapy itself cannot maintain long-term antitumor immunity, while the immune limitation by radiotherapy can be alleviated by blocking the PD-1/PD-L1 axis (130). On the other hand, the presence of immunosuppressive cells such as Tregs, myeloid-derived suppressor cells (MDSC) and anti-inflammatory macrophages in TME will provoke resistance to anti-PD-1/PD-L1 therapy (131). Radiation therapy can kill cancer cells while triggering the release of pro-inflammatory mediators and increasing tumor-infiltrating immune cells, in other words, transforming immune “cold” tumors into “hot” ones, thus enhancing the efficacy of immunotherapy and improving the side effects and resistance of immunotherapy through the development of combination therapy regimens, and broadening the limits of immunotherapy.

### 5.2 Methods to enhance the efficacy of CRC radioimmunotherapy

Anti-PD-1/PD-L1 drugs are useful when cancer cells or Treg release large amounts of PD-1. Treg upregulation of PD-1 is in response to more infiltration and proliferation of NK cells and CTL. This may start a few hours after stereotactic body radiation therapy (SBRT). However, it has been reported that peak upregulation of PD-1 can occur a few days after tumor irradiation, after which PD-1 expression decreases. This response suggested that anti-pd-1 should be started as soon as possible during SBRT and continue for a few days (or possibly longer) after radiotherapy. Anti-CTLA-4 is useful both before and after SBRT; use of anti-CTLA-4 can reduce the effect of Treg on CTL, so that administration before radiotherapy increases immunogenicity, while administration after radiotherapy attenuates the depletion of antitumor immunity (132). The regulation of immune checkpoints such as PD-1, CTLA-4, TIM-3 and TIGIT is highly dependent on tumor type. Therefore, it is necessary to consider tumor genetic factors when selecting the optimal targets and to study their temporal response to SBRT.

Among the different radiotherapy techniques, SBRT is the best choice for inducing abscopal effects (133). Because it induces abscopal effects and has minimal stimulatory effects on tumor-promoting cells including M2 macrophages, Treg and MDSCs. Immune checkpoint inhibitors (ICI) have high efficacy in metastatic colorectal cancer (mCRC) with microsatellite instability (MSI), but are ineffective in microsatellite stable (MSS) tumors due to low tumor mutational load. Selective internal radiation therapy (SIRT) enhances neoantigen production, which triggers a systemic antitumor immune response (i.e. abscopal effect) (134).

The detailed mechanism of the distal effect induced by the combination treatment is not yet clear. Consequently, treatment protocols should be context-specific to maximize efficacy (114). Clinically, radiotherapy is being explored in combination with a plethora of immune-based therapies to optimize anti-tumor immunity (135). Enhanced type I IFN production, cGAS-STING signaling activation or the use of IR in combination with several other therapies can enhance anti-tumor immune responses (136).

As the study progressed, the combination of RT with anti-CTLA-4 and anti-PD-1/PD-L1 is used to achieve optimal therapeutic results. Researchers constructed three mouse models of metastatic tumors in melanoma, breast cancer and pancreatic cancer. The results showed that anti-CTLA-4 initiated inhibition of Treg cells to expand the CD8/Treg ratio, and anti-PD-1/PD-L1 mainly increased the proportion of CD8+ TIL, but higher responses were obtained only with the involvement of RT, which diversified the T-cell receptor (TCR) of unirradiated tumor TIL (137).

Nanomedicine adjuvant technology introduces nanomedicines with optimized design to ameliorate the problem of low response rate and toxicity of cancer radioimmunotherapy, which are prepared by incorporating tumor antigens, immune or radioimodulators or biomarker-specific imaging agents into the corresponding optimized nanopreparations. This will help induce various biological effects such as generating *in situ* vaccination, promoting immunogenic cell death, overcoming radioresistance, reversing immunosuppression, and pre-stratifying patients and assessing treatment response or treatment-induced toxicity (138).

Since intestinal flora plays an important role in CRC tumor development and has some similar mechanisms and pathways of action as radiotherapy and immunotherapy. Thus, we will focus on the role of intestinal flora in the radiotherapy of CRC patients as follows.

### 5.3 Possibility of microbiota to improve the efficacy of radioimmunotherapy in CRC

The current cumulative evidence from CRC patients and animal studies has demonstrated a strong association between the gut microbiota and CRC. Enrichment of oncogenic flora not only elicits highly heterogeneous proliferation to form CRC, but also promotes tumor metastasis and drug resistance (139). Remarkably, gut microbiota can modulate both non-specific and specific immune functions in the body, which in turn affects tumor development as well as anti-tumor immune function. Bacteria can promote the transfer and cross-presentation of processed tumor antigen peptides in DC cells, reduce the frequency of CD4+CD25+ Treg cells and collectively promote T cell immunity (140). Bacterial constituents may also influence immunotherapy in CRC. For example, LPS is an outer membrane component of gram negative bacteria with abundant hydroxyl groups and some amide groups. Low doses of LPS are expected to be ideal stimulants for immune initiation (141). Furthermore, it has been demonstrated that there is a bi-directional interaction between the intestinal microbiota and radiotherapy. The gut microbiota can affect the anti-cancer clinical efficacy of radiotherapy, whilst ionizing radiation can alter the components and functions of the intestinal microbiota, which can lead to the development of radiation enteropathy. A potential mechanism for the abscopal effect-cGAS-STING signaling pathway can also be stimulated by the immunogenicity of microflora. Some reports suggest that bacterial DNA can activate the cGAS-STING pathway and upregulate type I interferon, which is a key cytokine for innate and adaptive immunity, resulting in an adjuvant anti-tumor immune response (142, 143). The above prompts us to speculate what role intestinal flora plays in radiotherapy, immunotherapy and radioimmunotherapy in CRC patients.

We speculate that there are two possible scenarios when gut microbiota are involved in combined radiation and immunotherapy and produce benefits: ① The gut microflora may be a bridge between radiotherapy and immunotherapy. Ionizing radiation causes changes in the environment in which the microbiota is located, which in turn promotes anti-tumor immunity and enhances the effects of immunotherapy. This suggests that the microbiota may act as a target for enhancing radioimmunotherapy. ② Although the gut microbiota is not a bridge, the outcome of microbiota therapy to enhance the efficacy of radiotherapy and immunotherapy is easy to guess due to the respective relevance of the microbiota to radiotherapy and immunotherapy. It may happen that modulation of the microbiota increases the efficacy of both radiotherapy and immunotherapy or only one of them. Furthermore, as the toxicities associated with radiation and immunotherapy are also important in the prognosis of CRC patients, modulating the microbiota is also expected to reduce the toxicities of both or either. In conclusion, the general principle is to improve both patient outcomes and prognosis, as well as to focus on prognostic quality of life.

Therapies for CRC targeting microbiota are constantly being updated and developed, including selective elimination of oncogenic microorganisms, lipopolysaccharide-promoted immunotherapy and targeted phage therapy (144). In addition to therapies targeting the intestinal microbiota itself, we observe that anti-tumor therapies mediated by bacteria as vectors have captured widespread attention because of their natural tumor-targeting ability and multiple immune-activating properties. For instance: due to its unique anaerobic properties, attenuated Salmonella typhimurium exhibits inherent tumor-specific colonization with little retention in normal organs and good biosafety (145). Today, the majority of CRC patients are of the pMMR/MSS phenotype who usually fail to receive satisfactory results after ICB therapy. It is a pity that the current general response rate to clinical immunotherapy is still low (20-30%) (146). Surprisingly, a recent study found a significant increase in PD-L1 expression in distal tumors treated with ^131^I-VNP, which may be related to the production of ev (i.e. extracellular vesicles, which play an important role in various intercellular communication processes) and stimulation of increased interferon by tumor cells after effective IRT. This implies that the immune checkpoint inhibitor αPD-L1, when promptly administered, may improve the immune response rate and produce a better immunotherapeutic effect on the immune response rate of ^131^I-VNP treatment. Moreover, the ^131^I-labeled attenuated Salmonella vector can also utilize the strong cytophilic activity of bacteria to eliminate primary tumors, and the DNA fragments produced by bacteria and IRT activate the cGAS-STING pathway to produce a large number of anti-tumor cytokines, providing an anti-tumor immune response for innate immunity. Also, tumor-associated antigens produced by the bacterial vector itself and ^131^I-VNP can significantly promote the maturation of DC cells, providing a basis for activating an adaptive anti-tumor immune response (147). Beyond the above strengths, compared with small nanomaterials, radiolabeled bacteria can be effectively retained at the tumor site for a long time, which can prevent tumor recurrence by inducing long-term immune memory effects, so as to achieve efficient IRT, reduce tumor recurrence rate and improve the quality of patient survival.

Besides, by studying the mechanism of gut microbiota in the development of CRC, we can also develop new anti-tumor targets and provide new ideas for new cancer treatment methods (148).

## 6 Summary and prospect

According to the above studies and discussions, we conjecture that based on the special role and close association of intestinal flora in the development of CRC, microbiota may act as a bridge to delicately connect radiotherapy and immunotherapy. Microbiota might act as an immunostimulant or immunomodulator to target the immune system of patients and thus influence the efficacy of immunotherapy, radiotherapy and their combination therapy. In particular, based on the fact that modulation of gut microbiota, such as FMT, possibly leads to a reduction in the incidence and severity of radiation enteritis and immune-related adverse events, gut microbiota may also be a common biological target for reducing the side effects of radioimmunotherapy, and how inhibition of this target to improve efficacy would also provide a positive direction for CRC patients to attain a longer survival and a higher quality of life after treatment. The above conjectures provide enlightening ideas for radioimmunotherapy mediated by bacterial pleiotropic immune activation functions. Novel interventions focusing on microbiota, such as bacterial engineering, next-generation probiotics, microbial-specific bactericidal antibiotics and fecal microbiota transplantation as monotherapies or add-on therapies, are promising for improving the efficacy of radioimmunotherapy.

Transforming conjecture into reality requires answering numerous outstanding questions, including the detailed mechanisms by which the microbiota modulates CRC and its associated therapies and requires insight into how the microbiota mediates the tumor microenvironment - either through direct effects on DNA damage and inflammation, or through other host-derived mechanisms. Fortunately, technological advances have provided us with revised tools to study the microbiota in the context of the growing number of physiological CRC model systems to decipher the challenging complexity of the colonic tumor microenvironment. We look forward to more breakthroughs in CRC genomics, metabolomics and immunology and hope that more experimental studies and clinical trials will follow to confirm these suspicions.

## Author contributions

HY, RG, ZW, FF and HZ all contributed to the writing and editing of the manuscript. All authors contributed to the article and approved the submitted version.
